# Supplementary figures and images for: Identification of the hub genes in gastric cancer through weighted gene co-expression network analysis
Source: PeerJ. 2021 Mar 5;9:e10682. doi: 10.7717/peerj.10682 (PMC7938783; doi:10.7717/peerj.10682)

### Sample dendrogram and trait heatmap

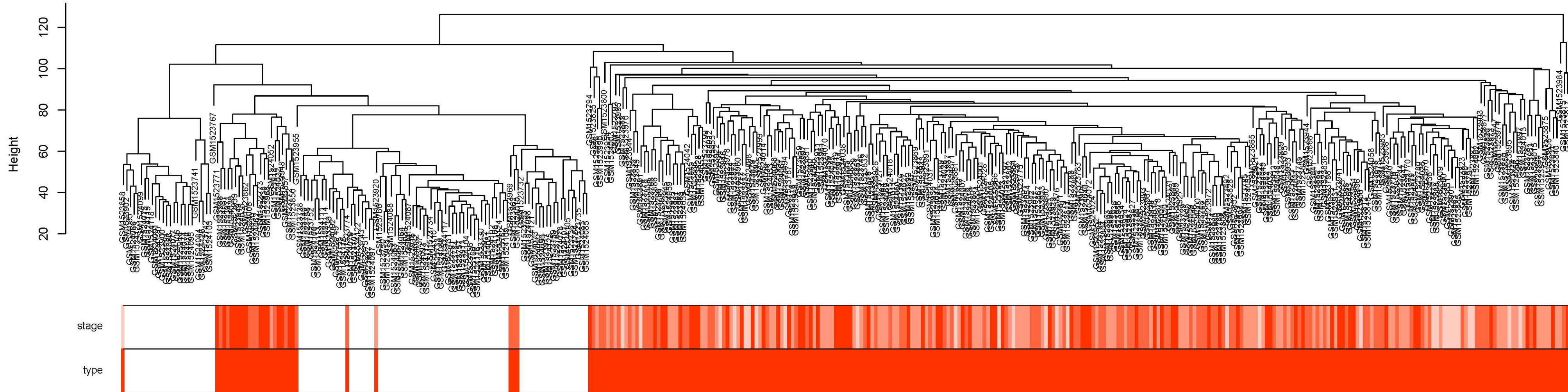

Supplement: Supplemental Information 1 — Clustering dendrogram of samples with a color indication of the trait type (normal=white, tumor=dark red), and stage (white represented normal samples, from light red to dark red designated as stage I to IV, respectively). The dendrogram shows no obvious outliers. [file peerj-09-10682-s001.pdf]
